# Supplementary material for: Femtosecond photoexcitation dynamics inside a quantum solvent
Source: Nat Commun. 2018 Oct 1;9:4006. doi: 10.1038/s41467-018-06413-9 (PMC6167364; doi:10.1038/s41467-018-06413-9)
Supplement: Supplementary file 1 — Supplementary Information [file 41467_2018_6413_MOESM1_ESM.pdf]

Supplementary information for

Femtosecond photoexcitation dynamics inside a quantum solvent

Thaler et al.

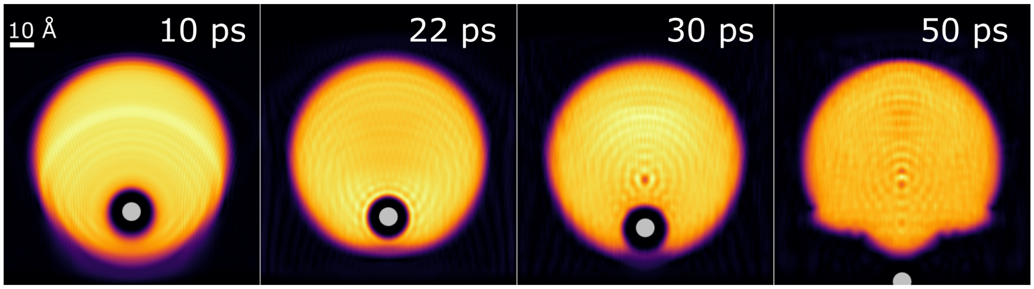

Supplementary Figure 1: Snapshots from Supplementary Movie 2. Helium density distributions of a He_{4000} droplet with an indium atom, originally located at 20 Å distance to the centre, as obtained with TDDFT for characteristic time delays. The dopant is ejected from the droplet after 60 ps, accompanied by a contraction of the solvation shell (bubble) around 22 ps.

Supplementary Note 1: The In-He_N excitation spectrum

The In-He_N excitation spectrum in the region of the In $5s^2 6s \leftarrow 5s^2 5p$ transition was previously recorded and is shown in Supplementary Fig. 2. In addition to the monomer signal (blue line) an In dimer band (red line) appears with strong overlap to the monomer. The monomer signal shows a maximum at 368 nm, which is blue-shifted by 2800 cm^{-1} with respect to the free atom line (green, solid line) [1]. The excitation wavelength was chosen at 376 nm (black, dashed line) to obtain a good monomer-to-dimer ratio. Additionally, a reduced pickup temperature was used to minimize the dimer influence.

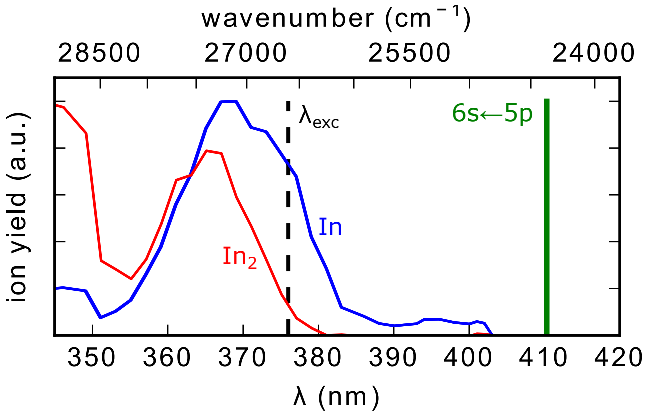

Supplementary Figure 2: Excitation spectrum of the indium monomer (In) and the indium dimer (In_2), both solvated inside He_N. The spectra are measured in a pump-probe experiment with 200 ps time delay and photoion detection at the In monomer mass (115 amu) and the In dimer mass (230 amu), respectively. The pump-probe delay time is sufficiently long that both monomers and dimers are ejected from the droplet and ionized in the gas phase. The spectra were recorded at a higher In pickup temperature as the presented experiments in order to obtain a stronger dimer signal. Additionally, the gas phase In transition (green, solid line) and the applied excitation wavelength (black, dashed line) are indicated.

Supplementary Note 2: The In-He pair potentials

The most important inputs for both the static and time-dependent DFT simulations are the dopant-helium diatomic potential energy surfaces for all electronic states that are populated in the experiment. The spin-orbit coupling corrected energy curve of the ground state ($X^2\Pi_{1/2}$), the first excited state ($2^2\Sigma_{1/2}$) and the ionic state of the In-He molecule are shown in Supplementary Fig. 3. All three states are spherically symmetric and the spin-orbit splitting of the ground state to the $^2P_{3/2}$ ($1^2\Pi_{3/2}$ and $1^2\Sigma_{1/2}$, not shown in Supplementary Fig. 3) has a value of about 2000 cm^{-1}, for which reason the latter is not taken into account in the simulation.

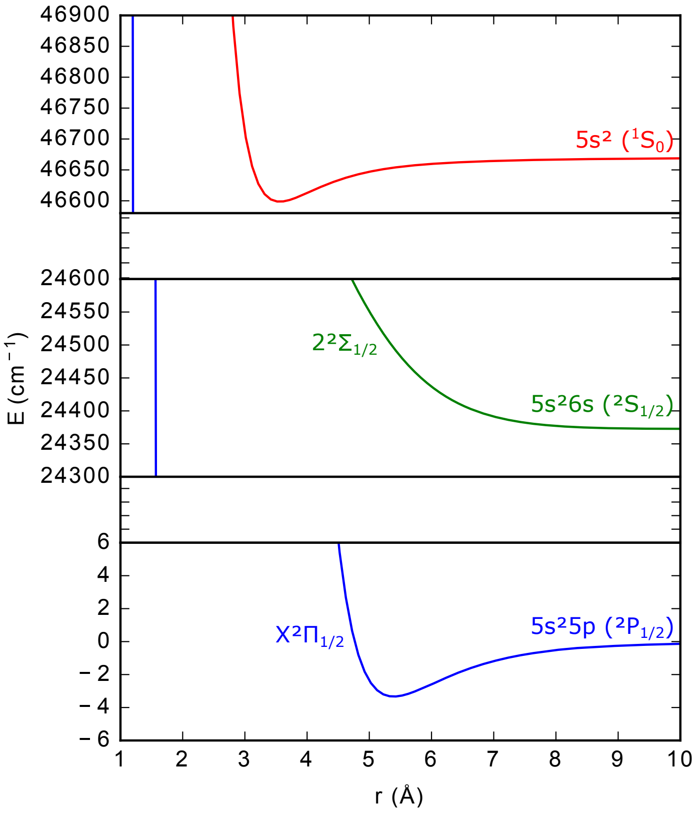

Supplementary Figure 3: Indium-Helium interaction pair potentials used for the DFT and TDDFT simulations. Ground state (blue), first excited state (green) and ionic state (red).

For completeness, we give a short summary of the ab initio strategy: In the calculation, the aug-cc-pV5Z family of basis sets [2, 3] in combination with the ECP28MDF effective core potential of the Stuttgart/Köln group [4] is used. The ab initio calculations are performed with the MOLPRO software package [5]. To account for the weak van der Waals-type binding between the He and In, a combination of multiconfigurational self consistent field calculations (MCSCF) [6, 7] and multireference configuration interaction (MRCI) [8, 9] is applied. The active space of the MRCI approach

consists of three valence electrons, the core orbitals are optimized in the MCSCF calculation and kept doubly occupied. The curves are basis set-extrapolated by applying additional calculations with the aug-cc-pVQZ and aug-cc-pVTZ basis set families and the three-point extrapolation formula by Wilson and Dunning [10]. By using the Breit-Pauli operator, the spin-orbit splitting is calculated.

Supplementary Note 3: Numerical error tests for the simulation

The large amount of 270 meV excess energy coupled to the system in the photoexcitation process, which is represented as high blueshift of the in-droplet excitation wavelength with respect to the free atom line (see supporting figure 2), is connected to an equally high amount of excited state interaction energy $E_{\mathrm{He_N\text{-}In^*}}$ (figure 3b). This situation requires a careful choice of the simulation parameters in order to avoid numerical errors. For example, the He repulsion by the excited state electronic wave function of the In atom causes a strong increase in the kinetic energy of the He. A correct description of the He movement requires a fine grid size and small time steps, especially within the first few fs, where the acceleration is high. We test for numerical errors by calculating the transient change of $E_{\mathrm{He_N\text{-}In^*}}$ for different grid sizes and different time steps, as shown in Supplementary Figs. 4 and 5.

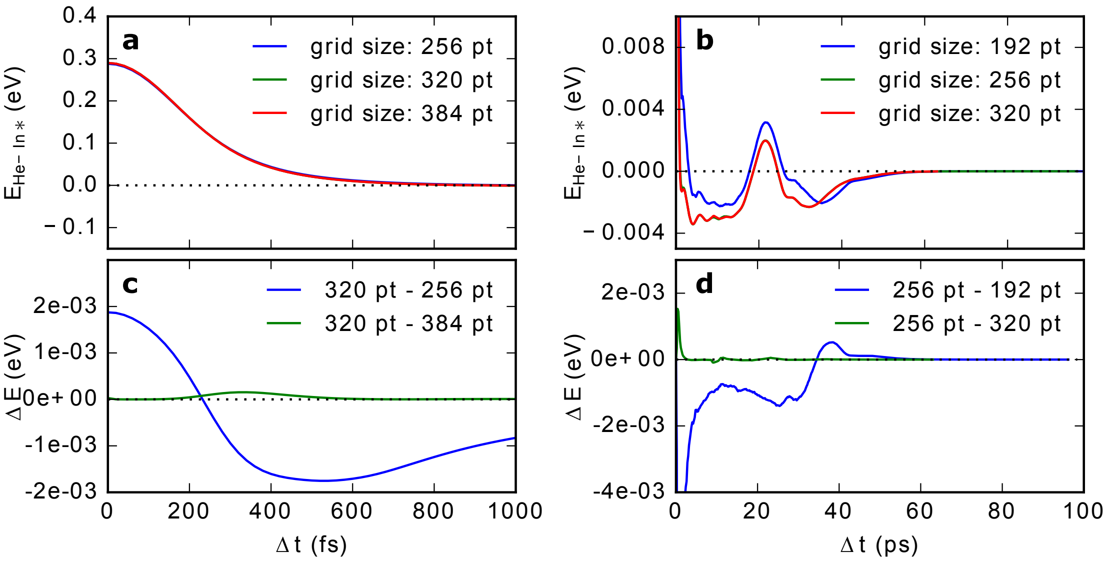

Supplementary Figure 4: Dependence of the excited state interaction energy $E_{\mathrm{He\text{-}In^*}}$ on the grid size parameters. (a) $E_{\mathrm{He_N\text{-}In^*}}$ as function of time within the first picosecond for three different grid sizes of 256, 320 and 384 pts, calculated with 0.10 fs time steps. (b) $E_{\mathrm{He_N\text{-}In^*}}$ for higher time delays for grid sizes of 192, 256 and 320 pt, calculated with 1 fs time steps. (c) Difference of interaction energy obtained with 320 pt grid size to that obtained with 256 and 384 pt, respectively. (d) Difference of interaction energy obtained with 256 pt grid size to that obtained with 192 and 320 pt, respectively.

The grid sizes used for the simulations presented in the paper are 320 pt for simulation of the bubble expansion dynamics (0 to 1 ps) and 256 pt to simulate the bubble oscillation (0 to 100 ps). Supplementary Fig. 4c shows that for the short dynamics an increase to 384 pt does not change the interaction energy significantly, while a decrease to 256 pt would introduce errors on the order of about 1%. A similar behavior is observed for the higher timescales (see figure 4d), where a grid size of 192 pt introduces numerical errors that are on the order of the simulated energies, whereas nearly no deviation to the grid size of 320 pt is found.

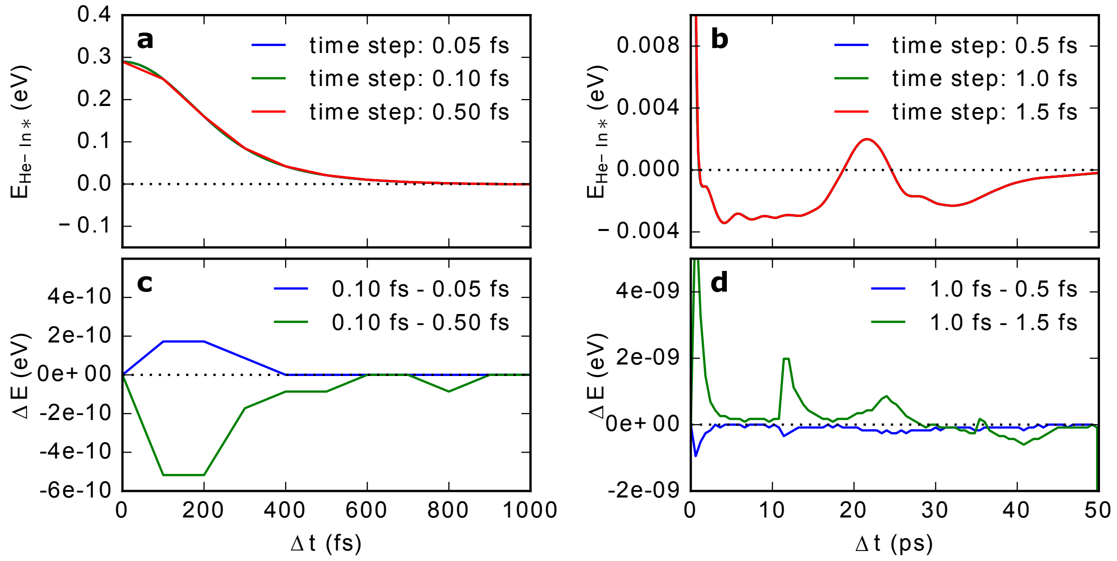

Supplementary Figure 5: Dependence of the excited state interaction energy $E_{\text{He-In}^*}$ on the time step parameter. (a) $E_{\text{He}_\text{N}\text{-In}^*}$ within the first picosecond as function of time for three different time steps of 0.50, 0.10 and 0.05 fs, calculated with a grid size of 320 pt. (b) $E_{\text{He}_\text{N}\text{-In}^*}$ for higher timedelays for the time steps of 1.5, 1.0 and 0.5 fs, calculated with a grid size of 256 pt. (c) Difference of interaction energy obtained with 0.10 fs to that obtained with 0.50 fs and 0.05 fs, respectively. (d) Difference of interaction energy obtained with 1.0 fs to that obtained with 1.5 fs and 0.5 fs, respectively.

The influence of the time step parameter turned out to be less pronounced, as shown in Supplementary Fig. 5c. An increase from 0.10 fs, as used for the shorter bubble expansion simulations, to 0.50 fs gives a slightly stronger change of $E_{\text{He}_\text{N}\text{-In}^*}$, as compared to a decrease to 0.05 fs, both of which are, however, in the 10^{-10} eV range. The influence on the simulation for the longer bubble oscillation (simulated with 1 fs steps) is an order of magnitude higher (see Supplementary Fig. 5d), but still remains in the 10^{-9} eV range.

Another important parameter is the cutoff-energy for the different pair potentials, that has to be chosen high enough in order to avoid unphysical He density cumulations and energetic instabilities. The cutoff-energies for the ground state, the excited state and the ionic state potential are chosen with 2150 cm^{-1}, 1008 cm^{-1} and 5560 cm^{-1}, respectively. As the excited state cutoff-energy has the lowest value, different energies around 1008 cm^{-1} were tested with the result that the influence on the excited state interaction energy was below 10^{-10} eV (not shown).

Supplementary Note 4: Bubble dynamics for different locations inside the droplet

Whereas the simulated bubble expansion dynamics at short time delays (<1 ps) show no dependence on the position within the droplet where the dopant is photoexcited, the ejection process and the accompanied bubble oscillation observed at longer time delays are strongly dependent on the photoexcitation position. In Supplementary Figure 6a the bubble radius over time for a starting location in the centre of the droplet is shown, revealing a continued oscillation of the solvation shell with a period of about 30 ps and no ejection. This is in contrast to the 20 Å off-centre excitation position, which shows only one contraction, superimposed to an overall increase of the radius due to the ejection. Supplementary Figure 6b shows calculated PE peak energies as function of delay time for photoexcitation at various distances to the droplet centre. The counter-propagating trends of bubble radius and PE energy for both the centre and the 20 Å position clearly show that a contracted bubble coincides with an increased PE energy, which is a consequence of the increased In-He interaction energy of smaller bubbles (see figure 1 in the main manuscript). Different appearance times of the first contraction for different starting locations can be explained with the superimposed PE energy decrease due to ejection, as well as effects caused by helium shock-waves that propagate through the droplet following the initial bubble expansion (see Supplementary Movies 2 and 3).

We choose the simulation of the 20 Å starting position for comparison with the measured transient PE peak shift (figure 3 of the main text) because for other locations either multiple or no bubble oscillations are predicted.

Supplementary Note 5: Transient ion yield and PE spectra at long time-delays

Ejection of the indium atoms can further be confirmed by a transient rise of ion yield. Because the ionic In^+-He potential is strongly attractive, the atoms deeply solvate into the droplets when being ionized within or even in the vicinity of the droplets, in which case they are not detected. Only when escaped from the long-range, attractive potential of the droplet they are truly free and are measured [11]. This is seen in the transient ion yield (Supplementary Figure 7a), where there is absent signal for the first 40 ps, followed by a signal rise within about 30 ps (to 67% of the maximum). The steady rise is connected to a position (and velocity) distribution of dopants inside the droplets before photoexcitation, resulting in an ensemble that gets ejected, which blurs the ion and electron transients. The same timescale of PE peak shift (figure 3 in main manuscript) and photoion yield rise confirms the correct interpretation of dopant ejection.

Further insight into the ejection process can be obtained from the line shapes of the PE lines. Supplementary Figure 7b shows PE spectra obtained from In–He_N at time delays of 0.8 ps (blue line) and 200 ps (red line), as well as for bare In atoms (yellow line). Ionization inside the droplet at 0.8 ps leads to a shift of the PE peak to higher energies with respect to the bare atom due to the reduced ionization potential inside the droplet [12]. The PE spectrum is significantly broader [(62 ± 2) meV, FWHM] compared to that of the bare atom [(35 ± 1) meV, FWHM] (see also figure 3

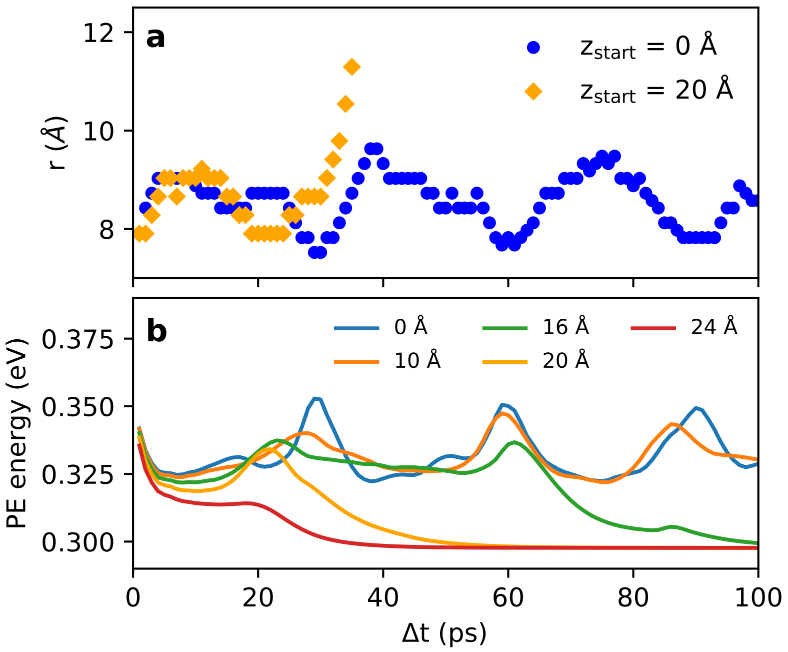

Supplementary Figure 6: Comparison of the simulated In-He_N dynamics for photoexcitation at different positions. The bubble radius as function of time is shown in (a) and the corresponding transient change in photoelectron energy is shown in (b).

of the main text), which we ascribe to the Franck–Condon overlap of the excited and ionic potential energy surface inside the droplet. Additionally, the 0.8 ps spectrum shows a wing extending below the bare atom line, representing decelerated electrons [12], as discussed in the results section of the main text. Ionization of the In–He_N system at 200 ps gives exactly the same line shape as the bare atoms, proving that all In atoms are ejected from the droplets.

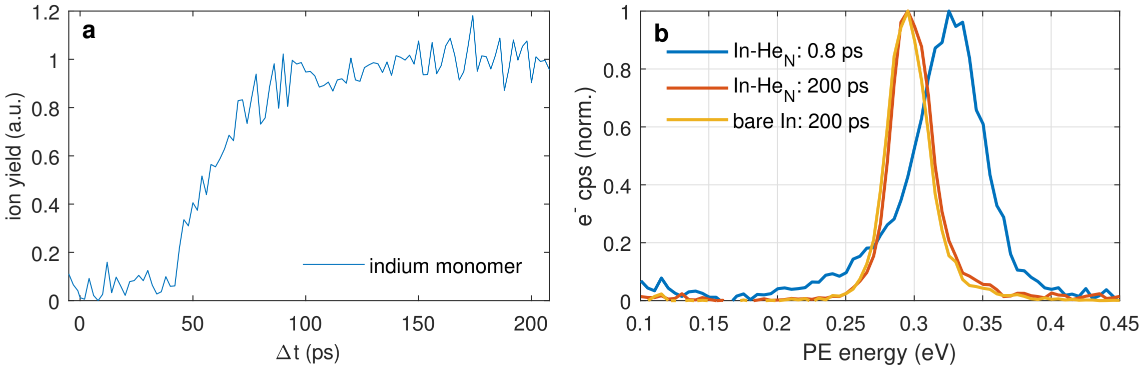

Supplementary Figure 7: Transient photoion yield (a) and comparison of PE spectra at short and long time delays, as well as a PE spectrum of bare In atoms (b). The bare atom spectrum was recorded by deactivating the droplet source and exploiting the effusive In atom beam from the pickup source. The excitation wavelength for the bare indium spectrum was chosen with 410 nm (see Supplementary Figure 2).

Supplementary References

1. Kramida, A., Yu. Ralchenko, Reader, J. & and NIST ASD Team. NIST Atomic Spectra Database (version 5.5.2), [Online]. Available: https://physics.nist.gov/asd [Fri Jan 19 2018]. National Institute of Standards and Technology, Gaithersburg, MD. 2018.
2. Woon, D. E. & Dunning-Jr., T. H. Gaussian basis sets for use in correlated molecular calculations. IV. Calculation of static electrical response properties. *J. Chem. Phys.* **100,** 2975–2988 (1994).
3. Peterson, K. A., Figgen, D., Goll, E., Stoll, H. & Dolg, M. Systematically convergent basis sets with relativistic pseudopotentials. II. Small-core pseudopotentials and correlation consistent basis sets for the post-d group 16–18 elements. *J. Chem. Phys.* **119,** 11113–11123 (2003).
4. Metz, B., Stoll, H. & Dolg, M. Small-core multiconfiguration-Dirac–Hartree–Fock-adjusted pseudopotentials for post-d main group elements: Application to PbH and PbO. *J. Chem. Phys.* **113,** 2563–2569 (2000).
5. Werner, H.-J. *et al. MOLPRO, version 2012.1, a package of ab initio programs* see http://www.molpro.net. 2012.
6. Knowles, P. J. & Werner, H.-J. An efficient second order MCSCF method for long configuration expansions. *Chem. Phys. Lett.* **115,** 259–267 (1985).
7. Werner, H.-J. & Knowles, P. J. An efficient internally contracted multiconfiguration reference CI method. *J. Chem. Phys.* **89,** 5803–5814 (1988).
8. Knowles, P. J. & Werner, H.-J. Internally contracted multiconfiguration reference configuration interaction calculations for excited states. *Theor. Chem. Acc.* **84,** 95–103 (1992).
9. Boys, S. F. & Bernardi, F. The calculation of small molecular interactions by the differences of separate total energies. Some procedures with reduced errors. *Mol. Phys.* **19,** 553–566 (1970).

10. Wilson, A. K. & Thom H. Dunning, J. Benchmark calculations with correlated molecular wave functions. X. Comparison with "exact" MP2 calculations on Ne, HF, H_2O, and N_2. *J. Chem. Phys.* **106,** 8718–8726 (1997).
11. Von Vangerow, J. *et al.* Imaging excited-state dynamics of doped He nanodroplets in real-time. *J. Phys. Chem. Lett.* **8,** 307–312 (2017).
12. Loginov, E., Rossi, D. & Drabbels, M. Photoelectron spectroscopy of doped helium nanodroplets. *Phys. Rev. Lett.* **95,** 163401 (2005).
