# Supplementary material for: Femtosecond photoexcitation dynamics inside a quantum solvent
Source: Nat Commun. 2018 Oct 1;9:4006. doi: 10.1038/s41467-018-06413-9 (PMC6167364; doi:10.1038/s41467-018-06413-9)
Supplement: Supplementary file 3 — Description of Additional Supplementary Files [file 41467_2018_6413_MOESM3_ESM.pdf]

Description of Additional Supplementary Files

File Name: Supplementary Movie 1

Description: Bubble expansion process. Time dependence of the helium droplet (HeN) density with an indium (In) atom located at the centre for the first two picoseconds after photoexcitation, as obtained from the TDDFT simulation. An expansion of the helium bubble is followed by a shock wave that propagates to the droplet surface, as can also be seen in Figure 1 of the main paper

File Name: Supplementary Movie 2

Description: Bubble contraction and dopant ejection. The time evolution of the helium density for the first 70 ps after photoexcitation with an indium atom placed at 20 ˚A from the centre. After the bubble expansion, the solvated atom is ejected from the droplet within about 60 ps. Additionally, a contraction of the bubble at around 22 ps during the ejection process occurs, indicating that one single period of the bubble oscillation can be observed.

File Name: Supplementary Movie 3

Description: Bubble oscillation. The time evolution of the helium density for the first 70 ps after photoexcitation with an indium atom placed directly in the droplet centre. Photoexcitation induces multiple oscillations of the solvation shell bubble. We note that the In atom appears to be at rest in the droplet centre because the simulation is based on zero initial velocity. In reality, a velocity of a few meters per second leads to translation to the droplet surface.
